# Endoscopic tympanoplasty with post-conchal perichondrium in repairing large-sized eardrum perforations

**DOI:** 10.1007/s00405-022-07476-7

**Published:** 2022-06-09

**Authors:** Chin-Kuo Chen, Hsin-Chiao Hsu, Min Wang

**Affiliations:** 1 Department of Otolaryngology-Head and Neck Surgery and Communication Enhancement Center, Chang Gung Memorial Hospital, Taoyuan, Taiwan; 2 Department of Otolaryngology-Head and Neck Surgery, Chang Gung Memorial Hospital, Keelung, Taiwan; 3 School of Traditional Chinese Medicine, College of Medicine, Chang Gung University, Taoyuan, Taiwan; 4 Department of Medical Education, Chang Gung Memorial Hospital, Taoyuan, Taiwan

**Keywords:** Post-conchal perichondrium, Tympanoplasty, Endoscopic ear surgery, Large-sized perforation, Temporalis fascia

## Abstract

**Purpose:**

This study aimed to compare the outcomes of endoscopic tympanoplasty with post-conchal perichondrium (PCP) and microscopic tympanoplasty with temporalis fascia in repairing large-sized eardrum perforations.

**Methods:**

We performed a retrospective chart review of 43 patients who underwent type 1 tympanoplasty for simple large-sized perforations. The patients were divided into two groups: Group I (endoscopic ear surgery with a PCP graft, 22 patients) and Group II (microscopic ear surgery with temporalis fascia, 21 patients). Graft success rate, demographic data, pre- and postoperative pure-tone average and word-recognition score, closure of the air − bone gap, and postoperative pain scale scores were compared between the two groups.

**Results:**

The graft success rates in groups I and II were 86.3% and 85.7%, respectively. The mean operation time in Group I (79.8 ± 16.5 min) was significantly shorter than that in Group II (99.9 ± 26.7 min) (*p* = 0.006). Both groups showed significant improvements in the pure-tone average and word-recognition scores. Average closure of the air − bone gap (ABG) in groups I and II was 20.7 ± 6.9 dB and 17.6 ± 8.4 dB, respectively. The reduction in ABG in Group I had a significantly higher magnitude than in Group II at 1000 and 2000 Hz, respectively (*p* = 0.028 and *p* = 0.017). The two groups showed no significant difference in postoperative pain scores.

**Conclusion:**

Endoscopic tympanoplasty with PCP showed a reliable, fascia-preserved, and excellent outcome in repairing large-sized perforations.

## Introduction

Tympanoplasty was developed to repair perforated tympanic membranes and is commonly performed via microscopic or endoscopic approaches. In comparison with transcanal endoscopic ear surgery (TEES), microscopic ear surgery (MES) sometimes requires soft tissue dissection, external skin incisions, external auditory canal (EAC) widening, and mastoid retraction for a better surgical view [1, 2]. Thus, TEES is more popular in middle ear surgery, and it offers the advantages of a reduced need for canalplasty, an extended operative field that can facilitate identification of hidden structures, and better visualization of the anterior meatal angle, which is crucial for repair of large-sized or anterior perforations and avoids lateralization or anterior blunting [3–5].

The technical concerns and success rates of these repair procedures vary depending on the size of the perforations. Large perforations are associated with lower success rates and a higher risk of recurrence because of increased technical difficulty, a narrowed graft surface overlapping with residual tympanum, deficient graft fixation, poor vascular supply, and a larger area requiring vascularization and epithelialization [6, 7].

The temporalis fascia is the most widely used graft in tympanoplasty, although it may involve scant graft and dormant-site morbidity in revision cases. The perichondrium is of mesodermal origin, similar to fascia [8]. The tragal perichondrium is widely used for the transcanal approach in tympanoplasty because of its location and accessibility. However, due to its size limitation, post-conchal perichondrium (PCP) has been used as an alternative. The PCP is located at the auricle and is suitable for large-sized perforations and may maintain the integrity of the temporalis fascia, since it may require further operation in cases of recurrence or cholesteatoma. We had previously reported using PCP to repair a small-medium eardrum perforation with the pop-through method under a microscope, which yielded a success rate of 90.5% [9].

Many studies have reported the outcomes of using the temporalis fascia or cartilage to repair a large, perforated eardrum in tympanoplasty [10, 11]. The temporalis fascia has poor dimensional stability and may cause residual perforation, especially in large perforations [12]. The “cartilage island” and “palisade cartilage” techniques could strengthen the stability of the graft, but may worsen audiometric outcomes [12]. However, no previous report has described the use of PCP in large perforation repair or compared the take rates between TEES with PCP and MES with temporalis fascia. Therefore, the present study aimed to compare the outcomes of endoscopic tympanoplasty with PCP and microscopic tympanoplasty with temporalis fascia in repairing large-sized perforations in adults with simple otitis media.

## Materials and methods

### Patients

This retrospective comparative study included patients who underwent type 1 tympanoplasty for simple large-sized perforations. The patients were classified into two groups based on the surgical procedure performed: Group I, which included patients who underwent endoscopic ear surgery with a PCP graft, and Group II, which included patients who underwent MES with temporalis fascia. Tympanic perforation was classified using the approach described by Srinivasan et al. [13]. Patients who had a perforation larger than 50% of the pars tensa were enrolled in this study. Patients with fixed or dislocated ossicular chains, cholesteatomas, or middle ear pathology were excluded from the study. Patients with posterior overhang canal wall and an unclear annulus of the tympanomeatal (TM) flap were treated with limited canalplasty with a curette or a microdrill.

Patients who underwent MES from September 2008 to August 2011 were included in the microscopic group, while endoscopic ear surgery was performed from 2012 to 2018. To avoid technical bias, we did not enroll the patients who had undergone the procedures in 2011 because it represented the initial phase of TEES adoption at our hospital. All patients were admitted for a 3-day hospital stay and observed for possible complications. This retrospective study was performed in accordance with the Declaration of Helsinki and was approved by the Medical Ethics Committee of Chang Gung Memorial Hospital (IRB: 202000200B0C501).

### Surgical technique

#### Endoscopic tympanoplasty with post-conchal perichondrium

**Fig. 1:**
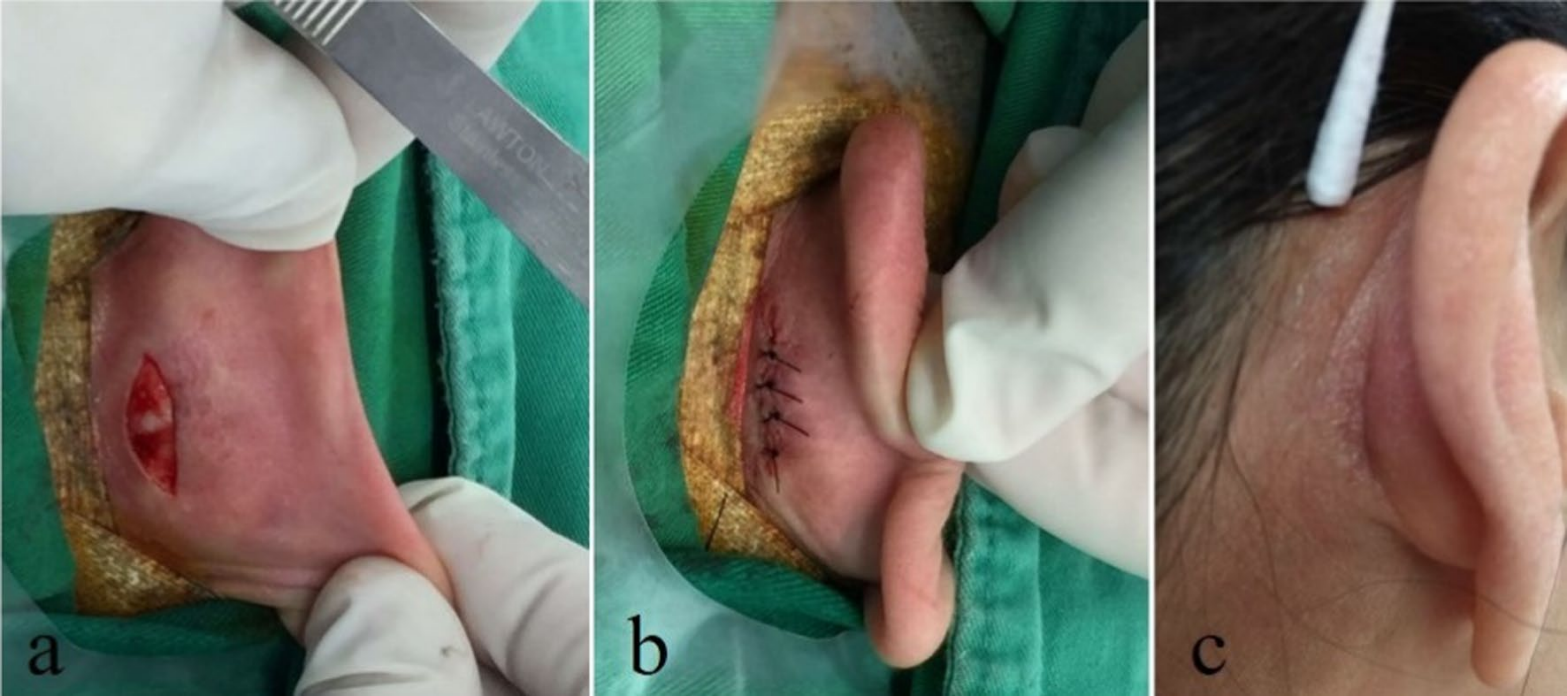
Harvesting the post-chonchal perichondrium. **a** The post-conchal cartilage exposed after a postauricular incision over the retroauricular surface. A surgical suture was then made near the post-auricular sulcus (**b**), and no visible scar remained with full healing after surgical suture removal (**c**)

All patients underwent tympanoplasty type 1, which was performed by a senior surgeon. An incision was made over the posterior auricle after local infiltration with epinephrine to minimize blood loss. The technique started with a postauricular incision to obtain a wider surgical view and to harvest PCP with approximate dimensions of 1.5 × 2 cm. Subsequently, the PCP graft was pressed on a plate to dry out. Attachment of the adhesive plaster enabled full exposure of the surgical field without hair shaving (Fig. 1). We used a rigid endoscope (diameter, 3 mm; 0° or 45°; length, 14 cm; Karl Storz, Tuttlingen, Germany) and a full HD monitor (Karl Storz, Tuttlingen, Germany). An antifogging liquid was used to avoid blurred vision. Next, 2% xylocaine with epinephrine was injected subcutaneously into the four quadrants of the outer ear canal for local anesthesia and water dissection. After denuding the margin of eardrum perforation by a Rosen needle or an alligator, the TM flap was elevated to reveal the middle ear cavity. Fibrous bends or granulation over the ossicles, promontory, E tube orifice, and ventilation routes, especially around the isthmus, were removed if present. Ossicular chain mobility was assessed intraoperatively. A dry PCP graft was trimmed to an appropriate size and placed medial to the malleus handle, the margin of the perforation, and the TM flap. If the tympanic membrane detached from the malleus during the surgery, the PCP was placed over the malleus handle and under the annulus using an over-underlay technique. The Gelform was placed into the middle ear cavity to support graft PCP, and the TM flap was repositioned. Finally, the EAC was packed with Gelform soaked in antibiotics. All patients underwent surgery using the transcanal approach.

#### Microscopic tympanoplasty with fascia

In Group II, a conventional microscope was used. After local anesthesia, the temporalis fascia was harvested and prepared for grafting. Surgery was performed using a retroauricular incision. The EAC was widened by a curette or microdrill during the surgery when visualization of the middle ear cavity was difficult due to anterior bony overhang. Tympanoplasty type I procedures were then performed. The retroauricular wound was closed, and a mastoid dressing was applied.

#### Study outcomes and measurement

All patients were followed up postoperatively at our outpatient department once a week during the first month before removal of the external ear packing, and then at 2, 3, and 6 months and at least 1 year postoperatively. The mean clinical follow-up durations in Group I and Group II were 388 and 365 days, respectively. We documented the status of the eardrums by using an endoscope or video-otoscope. Hearing thresholds, including air conduction, bone conduction, and mean air − bone gap (ABG), were calculated at 0.5, 1.0, 2.0, 4.0 kHz preoperatively and postoperatively. The graft take rate was defined as the percentage of the intact tympanic membrane without perforation that lasted for at least 1 year postoperatively.

The pure-tone average (PTA, in dB HL) and the word-recognition score (WRS, in %) were reported on the scattergram. We rounded the PTA calculated at the thresholds of 0.5, 1.0, 2.0, 4.0 kHz to the nearest whole number. WRS was measured using Chinese words presented at a 40-dB sensation level. PTA was plotted on the y axis of the scattergram in 10-dB intervals from 0 to 100 dB from bottom to top, whereas WRS was plotted on the *x* axis of the scattergram in 10% intervals from left to right. A postoperative scattergram was prepared with variations in PTA in ± 10 dB plotted on the *y*-axis with 0 dB at the middle point and with variations in WRS in ± 10% intervals with 0% at the middle point. We used the numeric rating scale (NRS 11) to evaluate postoperative pain intensity.

#### Sample size

G*power version 3.1 was used to determine the sample size [14]. Based on the preceding analysis with the effect size, a sample size of *n* = 21 in each group was needed to achieve a power of 95% at α = 0.05.

### Statistical analysis

Continuous data are expressed as means with standard deviation, while categorical variables are presented as counts with percentages in brackets. The Mann − Whitney *U* test was used to determine the correlation of clinical features between the two groups. Paired *t* tests were used to compare the pre- and postoperative audiometric parameters. Statistical significance was set at *p* < 0.05. All statistical analyses were performed using the Statistical Product and Service Solution (SPSS).

## Results

### Patient demographics

A total of 43 ears (14 men and 29 women) were included in the study. There were 22 patients in Group I and 21 patients in Group II. The mean postoperative follow-up periods were 420.5 ± 49.8 days in Group I and 411.6 ± 34.1 days in Group II. The two groups showed no significant differences in age at surgery, sex, postoperative follow-up duration, perforation type, perforation size, comorbidities (diabetes mellitus, hypertension, and coronary artery diseases), and preoperative symptoms such as dizziness, otalgia, otorrhea, tinnitus, and vertigo. The mean operation time in Group I (79.8 ± 16.5 min) was significantly shorter than that in Group II (99.9 ± 26.7 min; *p* = 0.006; Table 1). The graft success rate was 86.3% in Group I and 85.7% in Group II, and it showed no statistically significant difference between the two groups (*p* = 1.000).

**Table 1:** Baseline clinical and demographic parameters

	Group I (*n* = 22 ears)	Group II (*n* = 21 ears)	*p* value
Age at surgery, mean ± SD, yr	50.6 ± 13.5	45.9 ± 13.5	0.111
Follow-up period, mean ± SD, days	420.5 ± 49.8	411.6 ± 34.1	0.796
Sex, *n* (%)			0.526
Male	6 (27.3)	8 (38.1)	
Female	16 (72.7)	13 (61.9)	
Perforation type, *n* (%)			0.488
Central	20 (90.9)	21 (100.0)	
Marginal	2 (9.1)	0 (0.0)	
Perforation size, *n* (%)			0.536
50–75%	16 (72.7)	16 (76.2)	
> 75%	6 (27.3)	5 (23.8)	
Comorbidity			
DM, *n* (%)	2 (9.1)	2 (9.5)	1.000
HTN, *n* (%)	4 (18.2)	1 (4.8)	0.345
CAD, *n* (%)	4 (18.2)	1 (4.8)	0.345
Symptom			
Dizziness	1 (4.5)	2 (9.5)	0.607
Otalgia	1 (4.5)	2 (9.5)	0.607
Otorrhea	7 (31.8)	12 (57.1)	0.129
Tinnitus	8 (36.4)	3 (14.3)	0.162
Vertigo	5 (22.7)	3 (14.3)	0.698

*EES* endoscopic ear surgery; *PCP* post-conchal perichondrium; *MES* microscopic ear surgery; *SD* standard deviation; *DM* diabetes mellitus; *HTN* hypertension; *CAD* coronary artery disease

^*^p < 0.05, statistically significant differences between the groups

### Preoperative hearing presentation in groups I and II

**Fig. 2:**
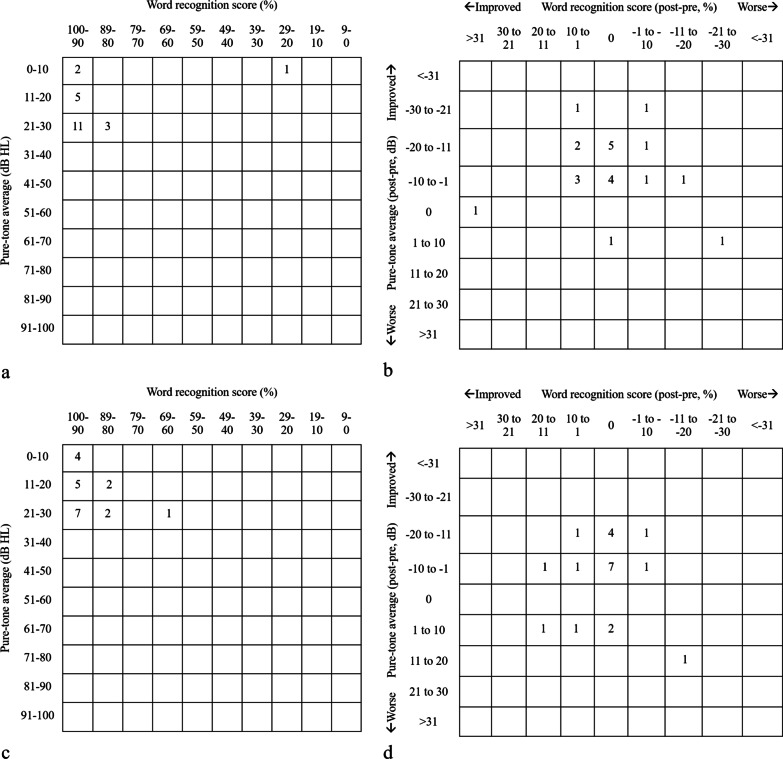
Scattergram diagrams of each group. **a** Preoperative pure-tone average and word-recognition score in Group I. **b** Postoperative air − bone gap closure and word-recognition score in Group I. **c** Preoperative pure-tone average and word-recognition score in Group II. **d** Postoperative air − bone gap closure and word-recognition score in Group II. *Post* postoperation; *pre* preoperation; *dB* decibel; *HL* hearing level

Preoperative PTA and WRSs of Group I and Group II are shown in Fig. 2a, c. The PTA of Group I was 42.4 ± 16.5 dB and that of Group II was 39.8 ± 12.5 dB (*p* = 0.705); the WRS of Group I was 92% and that of Group II was 95% (*p* = 0.284). The difference in preoperative bone conduction between Group I and II lacked statistical significance (*p* = 0.986), being 21.8 ± 14.6 dB and 22.6 ± 16.3 dB, respectively.

### Postoperative hearing presentation in groups I and II

Postoperative PTA and WRS improvements in comparison with the preoperative status in Group I and Group II are shown in Fig. 2b, d. Six patients in Group I and three patients in Group II showed improvement in both the PTA and WRSs. Nine patients in Group I and 11 patients in Group II showed improvement in the PTA, but did not show an improvement in the WRS. Four patients in Group I and two patients in Group II showed improvement in the WRS but did not show an improvement in the PTA. One patient each in groups I and II showed worse WRSs and PTAs postoperatively. The mean PTA improvement in Group I was 14.1 ± 7.9 dB and that in Group II was 8.5 ± 7.7 dB (*p* = 0.037). The WRS improvement was 2% in Group II and 1% in Group II. Both groups showed significant improvements in the PTA and WRSs. The postoperative bone conduction of Groups I and II was 18.2 ± 10.7 dB and 18.8 ± 17.9 dB, respectively (*p* = 0.559). There was no statistically significant difference between the two groups.

### Comparison of ABG closure after tympanoplasty in both groups

**Fig. 3:**
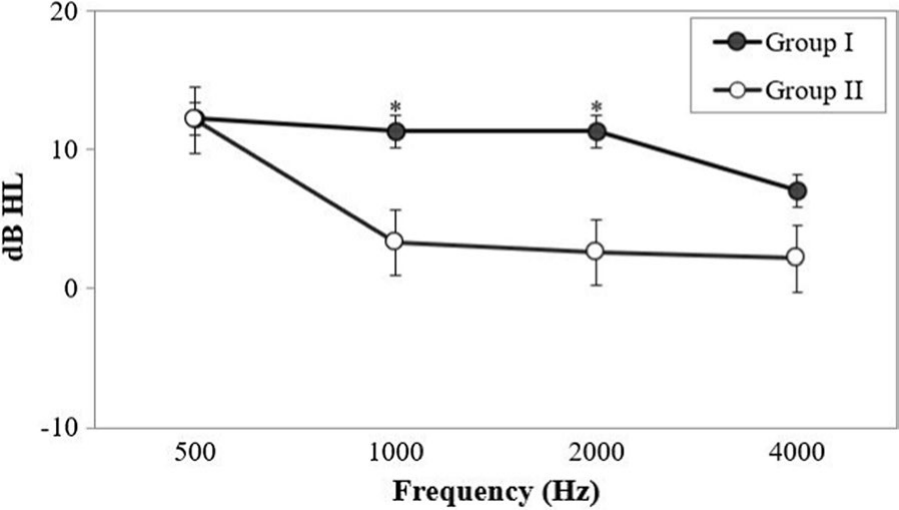
Comparison of ABG closure after tympanoplasty in the two groups. *p < 0.05, statistically significant differences between the groups. *ABG* air − bone gap; *dB* decibel; *HL* hearing level

Preoperative ABG in Group I and Group II was 20.7 ± 6.9 dB and 17.6 ± 8.4, respectively, without a significant difference (*p* = 0.164). Postoperative ABG in Group I and Group II was 10.2 ± 5.6 dB and 12.5 ± 9.4, respectively, again without a significant difference (*p* = 0.113). The mean reductions in ABG at each frequency in groups I and II are presented in Fig. 3. Both groups showed improvements at each frequency after tympanoplasty. No significant differences were observed between the groups at 500 Hz. The reduction in ABG in Group I was significantly better than that in Group II at 1000 and 2000 Hz. (*p* = 0.028 and *p* = 0.017).

### Postoperative pain assessment in groups I and II

**Fig. 4:**
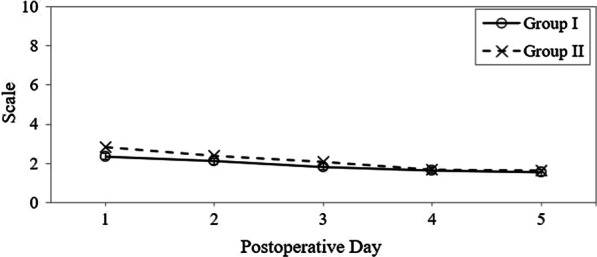
Pain numeric rating scale scores in both groups. Group I: endoscopic tympanoplasty with post-chonchal perichondrium. Group II: microscopic tympanoplasty with temporalis fascia

A numeric rating scale was used for postoperative pain assessment for 5 days, and the results are shown in Fig. 4. The pain scale scores in Group I were 2.4, 2.1, 1.8, 1.6, and 1.5, respectively, from day 1 to day 5, and those in Group II were 2.8, 2.4, 2.1, 1.7, and 1.6, respectively (*p* = 0.167, *p* = 0.416, *p* = 0.114, *p* = 1.000, and *p* = 0.996, respectively). The pain scale scores did not differ significantly between the groups.

## Discussion

Our study is the first to compare the outcomes of endoscopic tympanoplasty with post-conchal perichondrium versus microscopic tympanoplasty with temporalis fascia for repairing large perforations of the eardrums. The graft success rates were comparable: 86.3% in Group I and 85.7% in Group II. In addition, the operation time in Group I was shorter than that in Group II. Both groups showed significant improvements in the PTA and WRS. Group I showed greater closure of ABG than Group II at 1000 and 2000 Hz.

Various graft materials have been used to reconstruct large perforations, including temporalis fascia, perichondrium-reinforced cartilage palisade, full-thickness cartilage, and conchal cartilage reinforced by the temporalis fascia. The temporalis fascia is the most widely used graft material with a closure rate of 93%-97% in average-sized perforations [10] and is regarded as the gold standard for tympanoplasty [8]. Pradhan et al. reported graft uptake rates of 80% and 96.7% using fascia and full-thickness cartilage palisades, respectively, in patients with large perforations [11]. The success rate of tympanoplasty with temporalis fascia in repairing large-sized perforations was 75% as reported by Srinivasan et al., 64.7% by Alzoubi et al., and 71.4% by Atchariyasathian et al. (Table 2) [13, 15, 16]. Singh reported that the graft uptake rate using a 0.5-mm-thick sliced conchal cartilage reinforced by the temporalis fascia could be as high as 94.11% at the 6-month follow-up. The graft uptake rate was as high as 86.3% in Group I in our study, which is comparable to the findings obtained in Group II and with various other materials reported in other studies [10, 11, 17]. The majority of our patients showed significant improvement in the ABG (≥ 10 dB) and speech reception thresholds. At the 6-month follow-up, Gupta et al. observed that the postoperative pure-tone mean ABG was 12.67 ± 5.68 dB, in comparison with a value of 33.27 ± 4.29 dB preoperatively [10]. They reported that the average ABG closure at the 6-month follow-up was 11.67 ± 7.53 dB. Our results showed that Group I had better postoperative hearing gain and significantly better outcomes (*p* < 0.05). The bone conduction showed no difference between the two groups, both preoperatively and postoperatively. Thus, tympanoplasty with a PCP graft could achieve clinical success comparable to that with other graft materials, regardless of the technique used.

**Table 2:** Outcome comparisons in different published articles on repairing large-sized perforations

Author	Year	Case number	Duration of follow-up	Graft material	Approach technique	Surgical time (min)	Take rate
Pradhan [11]	2017	30	12 M	TF	ETT	–	80%
		30		Cartilage	ETT	–	96.7%
Srinivasan [15]	1997	40	6 M	TF	MTTM	–	75%
Alzoubi [16]	2010	32	6 M	TF	MTT	58.6	64.7%
Atchariyasathian [17]	2020	19	6 M	TF	ETT	100 ± 28	71.4%
The present study	2022	21	12 M	TF	MTT	99.9 ± 26.7	85.7%
		22		PCP	ETT	79.8 ± 16.5	86.3%

*TF* temporalis fascia; *PCP* post-conchal perichondrium; *ETT* endoscopic transcanal tympanoplasty; *MTTM* microscopic transcanal trans-tympanic myringoplasty; *MTT* microscopic transcanal tympanoplasty

PCP is worth considering in tympanoplasty for large perforations without postoperative complications, including chondritis, perichondritis, or reperforation in our study. PCP with an endoscopic approach offers several advantages over microscopic techniques using the temporalis fascia. First, our study revealed that the operative period in Group I was significantly shorter than that in Group II, while offering the advantage of minimal invasiveness. In comparison with MES, TEES can avoid canaloplasty and provide superior visualization as well as sufficient manipulation space, avoiding additional dissections and incisions and requiring shorter time for the management of soft tissue damage and evaluation of the middle ear [18]. Second, there was no need for ear dressing after endoscopic ear surgery, allowing patients to wear eyeglasses after surgery. Third, harvesting the temporalis fascia often requires extra dissection and requires a postoperative mastoid dressing, but not in a PCP grafting technique. In addition, patients in Group I had favorable cosmetic results (Fig. 1).

In our data, postoperative pain did not differ significantly between Group I and Group II, which was different from the results reported by Choi et al. The pain may have been caused by wound pain in Group I and craniofacial pain in Group II. Bhat et al. stated that most patients undergoing tympanoplasty with temporalis fascia experienced post-surgical pain and tenderness over the temporal region [19]. These feelings of discomfort at the harvest site occurred immediately after surgery and could occasionally persist for 3 months or more [19], while postoperative pain in Group I could recover over time.

The reperforation rate in cases with a perforation area > 50% was high, up to approximately 30% [7]. For revision cases requiring repeated temporalis fascia harvesting and cases requiring canal wall-down mastoidectomy for large grafts, craniofacial pain tends to be more severe than in primary cases [19]. Considering the high reperforation rate, patients with large perforations are recommended to receive PCP at first to reserve the temporalis fascia for reperforation repair due to future complications such as cholesteatoma, granuloma, and cochlear implant. PCP could also be suitable for revision surgery, in case of failure after tympanoplasty type 1 with other graft materials.

The limitations and drawbacks of the study are listed below. First, this was a retrospective study, and a summarization of the findings might be misleading because the surgeon's preference and patient's willingness determined the type of surgery in the study. Second, we recruited patients with simple chronic otitis media and excluded cases with cholesteatomas, middle ear pathology, and concomitant mastoidectomy. Confounding variables, such as the middle ear condition and the state of the perforation, may lead to different results. Third, even though the sample size was calculated, the number of patients included in the study was small. Further large randomized controlled studies with longer follow-up periods and multiple centers are needed.

## Conclusion

The results showed that the success rate in patients with large perforations in Group I was comparable to the results in Group II. Thus, endoscopic tympanoplasty is a good alternative to microscopic tympanoplasty since it provides a shorter operation time, minimal invasion, and a broader visual field. Owing to the high recurrence risk in patients with large perforations, PCP was suggested for initial repair because the temporalis fascia could be reserved for relapsed perforation or possible cholesteatoma. Because the graft take rate and audiometric results of tympanoplasty with post-chonchal perichondrium were comparable to those of other reconstructive materials, it can be a considerable choice for surgeons.
